# Effectiveness of Health Systems Strengthening Implementation Model to Improve Health of Pregnant and Lactating Women by Increasing Compliance with IFA and Calcium Tablets: A Quasi-Experimental Study

**DOI:** 10.3390/nu18142301

**Published:** 2026-07-14

**Authors:** Sutapa Bandyopadhyay Neogi, Mukesh Ravi Raushan, Sumesh Kumar, Ameet Babre, Nishtha Kathuria, Archana Mishra, Amiruddin Mohmmedmiyan Kadri, Harsh Bakshi, Nayankumar Popatlal Jani, Saloni Sidana, Jaya Chandra Reddy, Jennifer Hatchard, Manoj Raut, Tanuj Kaushik, Mini Varghese, Vismay Bharai, Minaxi Chauhan, Astha Vala, Shailesh Jagtap, Jennifer Busch Hallen, Jalal Choudhary

**Affiliations:** 1 International Institute of Health Management Research, New Delhi 110075, India; mukeshravi@iihmrdelhi.edu.in (M.R.R.); sumesh@iihmrdelhi.edu.in (S.K.);; 2 Nutrition International, Tara Crescent, Qutab Institutional Area, New Delhi 110016, India; ababre@nutritionintl.org (A.B.); nkathuria@nutritionintl.org (N.K.); msjcreddy@gmail.com (J.C.R.);; 3 National Health Mission (Madhya Pradesh), NHM Bhawan, T.T. Nagar, Bhopal 462003, India; 4 State Health Systems Resource Centre (SHSRC), Jivraj Mehta Bhavan, Gandhinagar 382010, India; 5 Directorate of Family Welfare, Sardar Patel Bhavan, Gandhinagar 382010, India; 6 Nutrition International, Ottawa, ON K2P 2K3, Canada; 7 Chief District Health Officer Office, District Panchayat, Vadodara 390001, India

**Keywords:** anemia, compliance, iron–folic acid, calcium, pregnancy, lactation, implementation research, India

## Abstract

Anemia affects nearly half of all pregnant and lactating women in India. Suboptimal compliance is often due to lack of awareness on effective management of perceived side effects, issues in ensuring regular last mile availability for distribution to beneficiaries owing to challenges in timely and adequate procurement supply chain management issues, family and household dynamics, lack of support for maternal nutrition. Implementation research was conducted in two states of India to deliver a package of interventions with the objective of preventing and managing anemia and improving health by increasing compliance with IFA and calcium supplements among pregnant and lactating women. **Method:** A quasi-experimental approach with a non-equivalent control group was adopted to assess the effectiveness of the model in a pragmatic setup across two blocks in each of the two states, divided into intervention and control arms (2007 pregnant and 2117 lactating women). Both arms continued with the standard government programs. Health systems interventions like supply chain strengthening, capacity building, behavior change were designed and delivered to ensure availability, accessibility and utilization of IFA and calcium by the beneficiaries in the intervention arm. The primary outcome was compliance with IFA among pregnant women. Secondary outcomes were compliance with calcium among pregnant women and IFA and calcium among lactating women. Difference-in-difference (DID) analysis was conducted, and logistic regression identified contributing factors. **Results:** The intervention package resulted in 26% improved compliance (DID: 8.1% vs. 1.5%) with IFA among pregnant women in the intervention arm compared to baseline. Similarly, compliance with IFA in lactating women increased by 49.2%. The attributable fraction was greatest (24.4%) for ‘proper instructions’ given. Compliance with calcium increased by 59% among pregnant women in one state, where the baseline parameters were lagging and interventions were initiated early within the stipulated time. Standardized ANC services and provision of proper instructions were consistently found to be associated with improved compliance. Interventions led to improved supply chain management, distribution of IFA and calcium in adequate quantities, increased awareness in the community, and better access to services. **Conclusions**: The intervention model was found to be effective in improving compliance with IFA in pregnant and lactating women. It is concluded that provision of proper instructions while distributing supplements seemed to have the greatest impact in improving compliance. State-specific variations highlight the need for tailored approaches. Scaling up such models, with emphasis on systemic and behavioral enablers, could strengthen India’s anemia control efforts.

## 1. Introduction

Anemia continues to be a public health problem affecting pregnant and lactating women across low- and middle-income countries [1,2]. In India, several health programs focus on the prevention and management of anemia. The flagship program of Anemia Mukt Bharat (AMB) is a testament to its commitment at the highest level. Iron deficiency is a leading cause of anemia, responsible for around half of anemia cases in women.

According to the AMB guidelines, the treatment for anemia in India includes daily iron and folic acid (IFA) supplementation (one tablet for prophylaxis and two tablets for therapeutic purposes) for at least 180 days during pregnancy (second and third trimesters). Each tablet contains 60 mg of elemental iron and 500 µg of folic acid. For lactating women, the same IFA dose is advised during the first six months after childbirth to rebuild depleted iron stores. In cases of severe anemia or intractable moderate anemia, intravenous preparations are recommended [1]. Emerging evidence from efficacy and effectiveness studies suggests that intravenous iron increases Hb levels slightly faster and reduces anemia in pregnancy compared to oral iron [3,4]. Intravenous iron may result in little to no difference in postpartum hemorrhage and blood transfusion rates [4,5].

Despite robust policy frameworks, program effectiveness has been hampered by poor compliance with supplementation. The issue of compliance is considered the key factor behind reduced effectiveness of oral iron across all age groups [6,7]. Gastrointestinal side effects, lack of information regarding side effects and their management, and long duration of treatment are reported to be key factors behind discontinuation of treatment [8,9].

It is reported that dietary intake of calcium supplements is less than 30% of recommended daily intake during pregnancy [10,11]. Calcium supplementation, which includes 500 mg of elemental calcium and 250 U of Vitamin D3 twice a day, during pregnancy is part of the national guidelines in India [10]. Calcium plays a crucial role in preventing hypertensive disorders in pregnancy, yet calcium supplementation uptake remains low. Supervision, monitoring, peer education, and family support are some effective ways of improving it [12]. Evidence shows that multi-component strategies, counselling on side effects, and reducing forgetfulness to consume tablets through family support or partner involvement could increase compliance [13,14].

Improving the health of pregnant and lactating women warrants prophylactic and therapeutic interventions to avert nutritional deficiencies. Distribution of supplements through health systems ensures wider population level coverage. However, it is hypothesized that compliance to treatment is fundamental for effective coverage not just distribution Given the persistent challenge of poor compliance, there is a growing need to test comprehensive, context-specific delivery models that can address both supply- and demand-side bottlenecks.

Nutrition International (NI) has been working in select geographies in India on IFA and calcium supplementation since 2016. Low compliance despite the improved distribution of supplements led to the genesis of this implementation research. It aimed to develop and implement an intervention model to improve compliance with oral IFA and calcium supplementation in pregnant and lactating women. The process involved formative research (for situational assessment to identify barriers and challenges qualitatively based on a health system framework), a population-based quantitative baseline survey (to quantify compliance), development of an implementation model, process evaluation during implementation, and an endline assessment. The components of the model were identified through formative research conducted at the start of the study [15], supplemented by inputs received from the state government and district authorities. The results indicated that despite strong political commitment and structured frameworks for anemia control, significant implementation gaps persisted: unclear guidelines at peripheral facilities, inconsistent testing methodologies, inadequate counseling on both content and methodology, occasional supply disruptions, overburdened staff with insufficient training, and limited mechanisms for tracking actual consumption. The model therefore comprised a package of health systems strengthening interventions to facilitate implementation of AMB activities in pragmatic settings.

An evaluation of the model was deemed necessary before scale-up. The objective of this manuscript is to explore the effectiveness of the intervention model in improving compliance with IFA and calcium supplementation in pregnant and lactating women.

## 2. Methods

### 2.1. Study Setting and Design

The implementation research was conducted in two states of India, namely Madhya Pradesh (MP) and Gujarat. These two states were selected based on purposive sampling, as NI was already providing technical assistance in these geographies to improve health system indicators. Two districts were selected based on composite indicators (derived from health, nutrition, and sociodemographic indicators) (Supplementary File S1). From each district, one high-and low-performing blocks (above and below the median) from each district were selected for the intervention. Blocks with similar profiles from the same district served as comparison, or control, blocks.

Design: The evaluation followed a quasi-experimental design with a non-equivalent control group. Population-based surveys were conducted by an independent third party agency in intervention and control areas for baseline and endline, using independent cross-sectional samples.

### 2.2. Interventions

Intervention arm: Based on the formative research findings [15], a package of interventions was designed to support availability, accessibility, and utilization of IFA and calcium by the beneficiaries. Prior to this study, NI engagement in Gujarat and MP consisted of technical assistance and advocacy support to state governments including budgeting, procurement, and monitoring/reporting. This implementation research represents the first instance of NI designing and testing a defined, multi-component model in a focused set of districts. The package comprised the following components: orientation and capacity building of frontline workers and supervisors on AMB guidelines; strengthening the supply chain through logistics training and follow-up to ensure last-mile availability of supplements; establishing model Village Health, Sanitation and Nutrition Day (VHSND) platforms to improve access and quality of maternal health services; community engagement and behavior change interventions using gender-responsive communication strategies; and policy and system-level advocacy to prioritize AMB implementation in planning and review mechanisms. NI, along with an implementation partner, provided continuous support through mentoring, field visits, and documentation across the intervention blocks. Additionally, routine health programs were implemented.

The control areas continued routine government programs without this additional support.

### 2.3. Outcomes

The primary outcome was compliance with IFA tablets among pregnant women. The case definition of compliance was consumption of at least 80% of tablets as appropriate for date, out of those received since the last visit within 15 days of the survey [5]. The 80% threshold was chosen to ensure comparability with previous studies, and a 15-day recall period was selected to minimize recall bias. Consumption was estimated based on self-reporting by pregnant and lactating women, further corroborated from empty blister packs shown at the time of the interview.

The secondary outcomes were the following:

- Compliance with calcium tablets among pregnant women, measured as consumption of at least 80% of tablets as appropriate for the date, out of those received since last visit (within 15 days).
- Compliance with IFA and calcium tablets among lactating women, measured as consumption of at least 80% of tablets as appropriate for the date, out of those received since last visit (within 15 days).

### 2.4. Variables/Indicators

**Figure 1:**
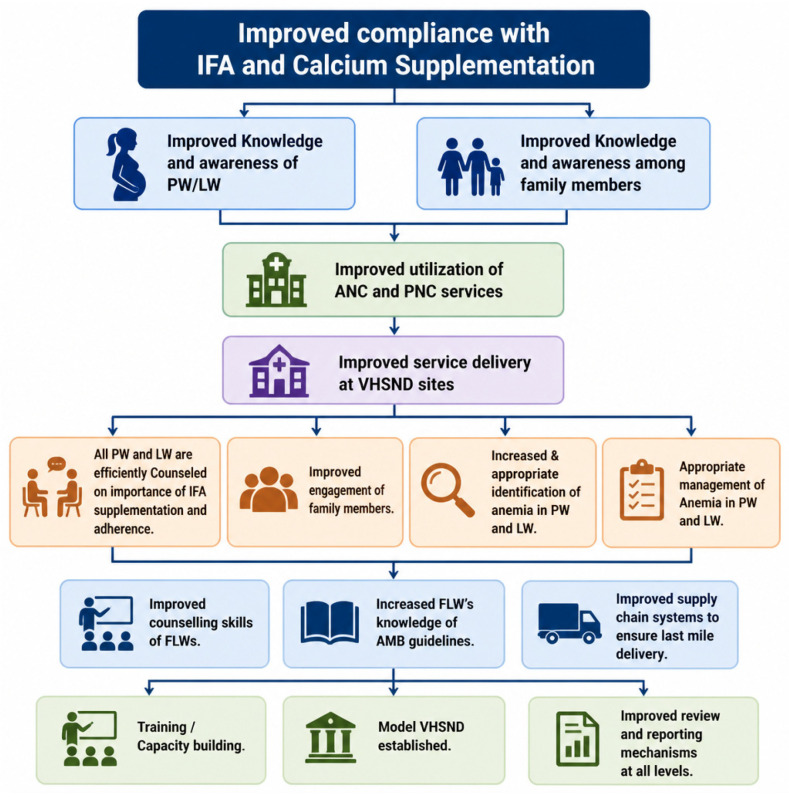
A conceptual framework to depict output indicators leading to outcomes (compliance with IFA and calcium) [PW—Pregnant women, LW—Lactating women, FLW—Frontline workers, ANC—Antenatal care, PNC—Postnatal care].

A Project Impact Pathway (PIP) was developed (Figure 1) to understand the process of changes leading to improved compliance (80% tablets consumed from total received) with IFA and calcium. Based on PIP, a conceptual framework is presented here to depict the output indicators with the outcomes. Appropriate indicators were identified to measure each of these outputs objectively.

### 2.5. Sampling and Sample Size

The baseline and endline surveys followed a multistage random cluster sampling technique.

Based on the prevalence of consumption of IFA tablets (at least 100 days during pregnancy) of 30.2%, an alpha error of 5%, a power of 80%, and a design effect of 1.5, the total number of beneficiaries was calculated to be 500 for each survey round (baseline and endline) and for each beneficiary category (pregnant and lactating women). Similarly, 150 field-level functionaries were required for each category.

From the list of primary health centers (PHCs) across blocks, one PHC from each block was selected randomly, proportional to the total number of households in villages covered by each PHC, using the Census 2011 Sampling frame. Cluster sampling was then used to select 25 villages from each PHC using Probability Proportional to Size (PPS). Systematic random sampling was used to identify a maximum of 5 pregnant and 5 lactating women from each village. The complete sample size and sampling strategy is described in Supplementary Table S1.

The total number of beneficiaries surveyed as part of the study is given in Table 1. The refusal rate was 0–2% in different categories.

### 2.6. Data Management

A structured data collection tool was developed by the research team in accordance with the study objectives. In addition to demographic and socio-economic factors, variables recommended in the PIP were incorporated into the tool. The tool was piloted and necessary modifications were made. Data were collected electronically by trained field staff using the CSPro database (version 7.0), a validated data management system with built-in checks. The data collection process was monitored daily in real-time by the core team remotely. Data were maintained in password-protected files. Every respondent was given a unique ID and confidentiality was maintained throughout. Data were analyzed using STATA version 17.

### 2.7. Statistical Analysis

The analysis was conducted using a serial cross-sectional approach, wherein the proportion of pregnant women compliant with IFA and calcium supplementation was computed for baseline and endline surveys separately, for both intervention and control sites for the two states. The difference in difference (DID) between intervention and control sites for baseline and endline surveys was also computed. Subgroup analysis was conducted by state.

Where results were statistically significant, bivariate analysis was performed to assess associations between compliance and intermediary variables (output indicators), followed by logistic regression.

We reported both unadjusted and adjusted odds ratio (OR) with 95% Confidence Intervals (CI). Output indicators where the adjusted OR was significant were selected to explore the association of process indicators with the final outcome (compliance).

Analysis was performed separately for the primary outcome (compliance with IFA in pregnant women) and secondary outcomes (compliance with calcium in pregnant women, and compliance with IFA and calcium in lactating women).

### 2.8. Ethical Considerations

The study was approved by the Institutional Review Board of IIHMR Delhi. Necessary permissions and approvals were obtained from the state governments and district authorities of both states. Written informed consent was obtained from every respondent before the start of the survey, and confidentiality was maintained throughout the process. Data were collected electronically, and anonymized data were transferred to the research team for cleaning and analysis. All data were stored securely, and only the core research team has access to them.

## 3. Results

The baseline survey was conducted in September–October 2022 and the endline survey in November–December 2024. The overall implementation period spanned 18 months. However, due to differences in administrative approvals and rollout timelines between the two states, the effective intervention period available for evaluation was shorter than the full implementation window: 12 months in MP and 8 months in Gujarat.

For both states combined, the compliance with IFA in pregnant women increased from 31.5% (baseline) to 36.5% (endline). A 26% improvement in compliance with IFA was observed in the intervention arm (Table 2). Factors associated with improved compliance included standardized ANC services (aOR 4.04; 95% CI 1.15, 14.1), supportive family members (aOR 2.71; 95% CI 1.15, 6.36), and proper instructions provided (aOR 1.63 to 2.63). The attributable fraction for comprehensive and standardized ANC services was 4.2%, and that of family support was 0.8%. Providing proper instructions to pregnant women on IFA and calcium intake, including information on perceived side effects, was attributed to 24.4% of the effect size (Supplementary Table S2 and Table 3). The above processes lead to improvement in ANC services that also included sensitization to pregnant women through IEC materials during VHSND sessions.

The intervention failed to demonstrate improved compliance with calcium during pregnancy at endline compared to baseline (baseline: 27.8% vs. endline: 24.3%). However, subgroup analysis indicated a significant 59% rise in MP (15.7% to 25.0%; *p* < 0.05), where baseline parameters were relatively low (Table 4). Factors leading to improved compliance in MP included good-quality ANC services (aOR 3.05; 1.79, 5.18), and proper instructions provided (aOR 1.96 to 3.3) (Table 5). Early initiation of interventions may have led to visible improvements within the study period. Compliance with calcium supplementation among lactating women was modest, at less than 5% overall.

The intervention resulted in a 49.2% improvement in compliance with IFA among lactating women (Table 6). Receipt of IFA tablets during postnatal visits contributed to improved compliance.

In both states combined, greater improvements were observed in programmatic activities in the intervention arm compared to the control arm between the baseline and endline surveys. This was evident in the proportion of women who were in regular contact with field-level functionaries, those tested for Hb, and those who received IFA and calcium in adequate quantities. The prevalence of severe anemia, as documented in their antenatal cards, also showed a decline (Supplementary Table S3).

## 4. Discussion

A health-system-strengthening intervention conducted in selected districts across two states in India demonstrated effectiveness in improving compliance with IFA among pregnant women (by 26%) and lactating women (by 49.2%). Standardized-quality antenatal services (attributable fraction 4.2%), supportive family members (attributable fraction 0.8%), and proper instructions provided during interactions with field-level functionaries (attributable fraction 24.4%) were associated with increased compliance. Improved compliance with calcium supplementation during pregnancy was observed in Madhya Pradesh.

The analysis showed improvement in compliance in both the control and intervention arms, resulting in a dilution of the ‘Difference in difference’ (DID) values. A deeper analysis of the intervention strategies revealed that advocacy efforts were undertaken at the district level, affecting all blocks. Although the interventions in both states were similar, some components had to be customized given the federal nature of the health system and to preserve the principles of implementation research. The effectiveness results were therefore more pronounced in one state where baseline indicators were relatively poor. Delays in obtaining approvals at varying time points, combined with the need to complete the study within a stipulated time frame due to resource constraints, resulted in varying intervention durations. This may have contributed to the differential results observed between the two states. The intervention spanned a period of 18 months, with different activities being initiated at varying time points. While most of the activities were implemented for a period of 12 months in MP, it was 8 months in Gujarat. Strategies on behavior change communication take a while to become institutionalized as they are implemented and undergo maturity with iterations. Although interventions over a period of 12 months can show improvement, long-term support is required for sustained benefits. In our study, contact with field-level functionaries (ASHAs at the village level) and their involvement in intervention activities was more frequent compared to ANMs (at subcenter level) and medical officers (at the PHC level). This may explain the relatively better performance of ASHAs than ANMs in facilitating compliance.

Evidence shows that nutritional interventions during pregnancy can improve fetal, neonatal, and infant outcomes [16]. However, the long-term direct effect of maternal nutritional interventions beyond the neonatal period remains a gap in evidence. The emerging literature suggests that, rather than individual micronutrients, balanced protein energy and lipid-based supplements may be more beneficial [17]. Optimal nutrition for long- term benefits can be obtained through the use of locally available foods with adequate macro and micronutrients during early pregnancy, supplemented by more comprehensive nutrient supplements where necessary [18]. The ongoing Anemia Mukt Bharat (AMB) program in India emphasizes at least four standardized-quality antenatal visits (or up to eight where feasible), with a focus on hemoglobin estimation and counselling on diet and nutrition . It clearly outlines the follow-ups required by field-level functionaries to maintain the channel of communication—essential for behavior change [1,2].

The term compliance is defined differently in the literature. While in some studies, compliance is defined as consumption of all prescribed tablets [19], others defined non-compliance as missing two or more doses in the last 7 days [20] or taking fewer than 100 tablets during pregnancy [21]. The definition used in this study was based on consumption of at least 80% of prescribed tablets for preventive or therapeutic purposes, as inferred from anemia status documented in the participant’s ANC cards, within the preceding 15 days only, to minimize recall bias. Additionally, effectiveness was assessed through serial cross-sectional surveys rather than a pre–post design. Any positive impact in such designs is more systemic and reflects the reach and adoption of interventions within the health system. This approach therefore strengthened the internal validity of our findings compared to similar pre–post designs.

Consistent messaging, regular reminders about the benefits of calcium, and ensuring adequate supply are some suggested measures to improve compliance [22,23]. Compliance with calcium supplementation was 27–30% in our study, which is comparable to existing evidence when a similar definition of intake of at least 80% of tablets is applied [24]. Adequate social and family support is known to enhance adherence to supplementation among pregnant women [25]. Furthermore, increased availability and access to supplements can enhance compliance [26].

Compliance is closely linked to the number of tablets required daily. Supplementation with IFA and calcium requires consumption of three to four tablets daily. Emerging evidence from some settings suggests that low-dose calcium is not inferior to high-dose supplementation [27]. Along similar lines, both daily and non-daily oral iron supplementation regimens effectively increase hemoglobin levels in patients with iron deficiency [9]. Reducing the number of tablets in the future is likely to improve overall compliance.

In our intervention, we introduced a WhatsApp-based Chatbot for field-level functionaries to plan and manage distribution of supplements. It was also used to provide beneficiaries with information on anemia and reminders of micronutrient supplementation as per the AMB guidelines. Mobile-based communication strategies are already in use for behavior change. Telephonic intervention is a client-friendly approach to boost compliance and mitigate side effects [28]. However, the effectiveness of these approaches remains limited [29].

The model was implemented within health system settings, largely by the health system functionaries, within the ambit of the AMB program, which is accorded priority at both national and state levels. The interventions catalyzed the implementation of approved activities that did not require major deviations from routine services and did not have additional financial implications. The layers of monitoring were kept to a minimum to adopt a more pragmatic approach, thereby enhancing its scalability.

The study has certain limitations. As the blocks were selected from the same district, a dilution of differences in effectiveness was observed. The interventions were introduced gradually in phases that followed different timelines in the two states, due to administrative approvals and operational constraints. This could have led to differential effect sizes between the states. The indicators to measure outputs overlapped, as is the case with any complex intervention, which precluded exploring the contribution of each intervention to the cumulative effect size. Grouping preventive and therapeutic interventions together in the data analysis was also a limitation. The determinants of adherence—including participant motivation, counselling needs, dosing regimens, duration of treatment, and follow-up requirements—differ between preventive and therapeutic anemia interventions. The decision to pool these groups was made because the AMB program operationalizes and monitors iron supplementation as a single continuum of care rather than as two distinct regimens. Furthermore, the intervention was designed to strengthen health system delivery processes, including supply, counselling, and tracking, that were common to both groups rather than targeting behaviors specific to either preventive or therapeutic supplementation. Consequently, the pooled effect estimate should be interpreted with caution, as it represents an average effect across two populations that may respond differently to the intervention.

Despite these limitations, the study provides sufficient evidence on the effectiveness of a complex intervention to improve compliance. The study followed a rigorous methodology with explicit case definitions and evaluation techniques. The study sites spanned two districts across states, representing different categories of health indicators, thereby strengthening external validity. The results were drawn from independent population-level surveys based on robust sampling methods and were independent of the intensity of implementation. The baseline and endline surveys were conducted in the same villages, thereby controlling for variables such as geographical location, distance from the district headquarters, skilled workforce, socioeconomic factors, etc. The interventions were directed at health systems strengthening, with scalability and sustainability as the prime focus.

## 5. Conclusions

A health-system-strengthening implementation model in selected districts has been shown to improve compliance with IFA among pregnant women by 26% and among lactating women by 49% in the intervention arm. Compliance with calcium supplementation during pregnancy improved by 59% in Madhya Pradesh. Nearly a quarter (24.4%) of the improvement was attributable to proper instructions provided to pregnant women regarding IFA and calcium intake. This cannot fully rule out confounding by related aspects of the interaction, such as time spent or communication style, which were not independently measured. Thus, instructions provided to pregnant and lactating women by well-informed and adequately supported field-level functionaries are essential for improving compliance. Improved communication with beneficiaries, interventions addressing supply chain management, and increased awareness through leveraging existing community-based platforms and channels are likely to contribute significantly to improving the health outcomes of pregnant and lactating women.

## Figures and Tables

**Table 1:** Number of respondents who participated in the study.

Type of Respondents	Baseline (2022)			Endline (2024)			Total		
	Control	Intervention	Total	Control	Intervention	Total	Control	Intervention	Total
Pregnant women	517	532	1049	436	522	958	953	1054	2007
Lactating women	519	534	1053	468	596	1064	987	1130	2117

**Table 2:** Effectiveness of the intervention on compliance with IFA in pregnant women in both states.

Indicator—IFA	Arm	Compliance at Baseline (%) (*n*)	Compliance at Endline (%) (*n*)	Difference (95% CI); *p* Value	Diff in Diff (95% CI); *p* Value
Both states		31.5 (*n* = 1049)	36.5 (*n* = 958)	5.0 (0.9, 9.2); 0.016	
-MP (Damoh)		21.6 (*n* = 528)	30.7 (*n* = 505)	9.1 (3.8, 14.4); 0.0008	
-Gujarat (Vad)		41.5 (*n* = 521)	43 (*n* = 453)	1.5 (−4.6, 7.8); 0.62	
Both states	Control	31.7(*n* = 517)	33.2 (*n* = 436)	1.5 (−4.4, 7.5); 0.61	−6.6 (−14.8, 1.7); 0.09
	Intervention	31.2 (*n* = 532)	39.3 (*n* = 522)	8.1 (2.3, 13.9); 0.006	
MP (Damoh)	Control	21.0 (*n* = 261)	24.9 (*n* = 253)	3.8 (−3.4, 11.1); 0.3	−10.7 (−21.2, 0.05); 0.05
	Intervention	22.1 (*n* = 267)	36.5 (*n* = 252)	14.5 (6.6, 22.1); 0.0003	
Gujarat (Vad)	Control	44.8 (*n* = 183)	42.6 (*n* = 256)	2.2 (−7.2, 11.7); 0.64	0.8 (−11.8, 13.3); 0.9
	Intervention	41.8 (*n* = 270)	40.4 (*n* = 265)	1.4 (−6.9, 9.8); 0.72	

**Table 3:** Logistic regression showing factors that influence compliance with IFA during pregnancy in both states (endline survey).

Indicators	Unadjusted OR (95% CI, *p* Value)	Adjusted OR (95% CI, *p* Value)
Respondents who were informed about anemia status (out of those in whom Hb was tested)	1.15, (1.14; 2.0), 0.003	0.65, (0.49; 0.88), 0.005
Respondents counselled about benefits of consuming IFA	1.46, (1.01; 2.11), 0.04	1.12, (0.73; 1.73), 0.577
Respondents counselled about dosage	1.66, (1.25; 2.22), <0.001	1.63, (1.19; 2.24), 0.002
Respondents instructed to consume the IFA tablet about 1 h after meal	2.59, (1.72; 3.93), <0.001	2.63, (1.6; 4.33), 0.001
Respondents instructed to consume IFA supplemented with lemon water	1.86, (1.29; 2.66), 0.001	1.32, (0.87; 1.99), 0.181
Respondents instructed to not consume IFA with calcium tablets	1.77, (1.11; 2.84), 0.016	2.17, (1.3; 3.64), 0.003
Respondents to whom no instructions were given	0.31, (0.10; 0.90), 0.032	0.91, (0.23; 3.51), 0.899
Respondents who knew signs and symptoms of anemia	0.75, (0.58; 0.99), 0.041	0.78, (0.56; 1.09), 0.157
Respondents who received more than 30 tablets in the last visit	1.72, (1.11; 2.64), 0.014	2.31, (1.39; 3.86), 0.001
Respondents who received care by family members (reminders about IFA and Ca, bought iron-/nutrient-rich food, support in heavy housework, etc.)	2.81, (1.23; 6.43), 0.014	2.71, (1.15; 6.36), 0.022
Respondents received services of ultrasound at ANC	1.73, (1.27; 2.36), 0.001	0.36, (0.1; 1.28), 0.117
Respondents received minimum package of ANC (weight, hemoglobin, blood pressure, ultrasound and blood sugar)	1.89, (1.39; 2.56), <0.001	4.04, (1.15; 14.1), 0.028
State	1.7, (1.3; 2.22), 0.001	1.35, (0.95; 1.92), 0.092

**Table 4:** Effectiveness of the intervention on compliance with Calcium in pregnant women.

Indicator: Calcium	Arm	Baseline (%) (*n*)	Endline (%) (*n)*	Difference (95% CI); *p* Value	Diff in Diff (95% CI); *p* Value
Both states		23.93 (*n* = 1049)	25.99 (*n* = 958)	2.06 (5.85; −1.73); 0.285	
-MP (Damoh)		16.48 (*n* = 528)	18.81 (*n* = 505)	2.33 (6.99; − 2.32); 0.325	
-Gujarat (Vad)		31.48 (*n* = 521)	33.99 (*n* = 453)	2.51 (8.43; −3.40); 0.403	
Both states	Control	23.60 (*n* = 517)	23.85 (*n* = 436)	0.25 (5.69; −5.17); 0.926	3.27 (−10.87; 04.32); 0.39
	Intervention	24.25 (*n* = 532)	27.77 (*n* = 522)	3.52 (8.83; −1.77); 0.192	
MP (Damoh)	Control	17.24 (*n* = 261)	12.65 (*n* = 253)	−4.59 (1.59; −10.78); 0.145	−13.86 (−23.12; −04.6); 0.003
	Intervention	15.73 (*n* = 267)	25.0 (*n* = 252)	9.27 (16.17; 2.37); 0.008	
Gujarat (Vad)	Control	30.08 (*n* = 256)	39. 34 (*n* =183)	9.26 (18.25; 0.28); 0.043	11.72 (−0.21; 23.6); 0.05
	Intervention	32.83 (*n* = 256)	30.37 (*n* = 270)	−2.46 (5.45; −10.37); 0.541	

**Table 5:** Logistic regression showing factors that influence compliance with Calcium during pregnancy in MP (endline survey).

Indicators	Unadjusted OR (95% CI, *p* Value)	Adjusted OR (95% CI, *p* Value)
Respondents counselled by ASHA	2.14, (1.18; 3.88), 0.012	1.96, (1.17; 3.27), 0.01
Respondents counselled about dosage	1.92, (1; 3.68), 0.048	1.98, (1.07; 3.67), 0.029
Respondents instructed to consume the IFA tablet about 1 h after meal	1.91, (1; 3.67), 0.049	1.62, (0.8; 3.28), 0.179
Respondents instructed to not consume the IFA tablet on an empty stomach	1.5, (0.86; 2.59), 0.145	0.9, (0.47; 1.7), 0.748
Respondents instructed to consume lots of water during the day	1.77, (1.06; 2.95), 0.028	1.07, (0.55; 2.07), 0.838
Respondents instructed to consume lots fruits and vegetables to avoid constipation	3.88, (2.22; 6.78), 0.0001	3.3, (1.65; 6.58), 0.001
Respondents instructed to consult doctors immediately if severe symptoms appeared	4.62, (1.68; 12.64), 0.003	3.0 (0.99; 9.08), 0.052
Respondents instructed to not consume IFA with calcium tablets	2.85, (1.54; 5.28), 0.001	2.29, (1.15; 4.54), 0.017
Respondents received services of ultrasound at ANC	2.6, (1.53; 4.42), 0.001	0.01, (0; 0), 0.987
Respondent received quality of services at ANC (weight, hemoglobin, blood pressure, ultrasound and blood sugar)	5.18, (1.79; 5.18), 0.001	3.05, (1.79; 5.18), 0.001

**Table 6:** Effectiveness of the intervention on compliance with IFA in Lactating women.

Indicator—IFA	Arm	Baseline (%) (*n*)	Endline (%) (*n*)	Difference (95% CI); *p* Value	Diff in Diff (95% CI); *p* Value
Both states		13.39 (*n* = 1053)	19.36 (*n* = 1064)	5.97 (2.82; 9.11) 0.0002	
-MP (Damoh)		11.1 (*n* = 529)	25.86 (*n* = 549)	14.7 (10.14; 19.28) 0.0001	
-Gujarat (Vad)		15.64 (*n* = 524)	12.43 (*n* = 515)	3.22 (−1.01; 0.74) 0.13	
Both states	Control	12.71 (*n* = 519)	17.31 (*n* = 468)	4.6 (−9.04; −0.14) 0.04	2.3 (−8.6; 3.9) 0.47
	Intervention	14.05 (*n* = 534)	20.97 (*n* = 596)	6.92 (2.48; 11.4) 0.001	
MP (Damoh)	Control	11.79 (*n* = 263)	22.58 (*n* = 279)	10.7 (−17.13; −4.46) 0.0009	8 (−17.1; 1.2) 0.08
	Intervention	10.53 (*n* = 266)	29.26 (*n* = 270)	18.7 (12.13; 25.3) 0.0001	
Gujarat (Vad)	Control	13.67 (*n* = 256)	9.52 (*n* = 189)	−4.15 (−9.59; 10.25) 0.183	0.73 (−9.337; 7.895) 0.87
	Intervention	32.83 (*n* = 268)	30.37 (*n* = 326)	−2.46 (5.45; −10.37) 0.541	

## Data Availability

The data will be made available on request and after due approval from the Government.

## Supplementary Materials

The following supporting information can be downloaded at: https://www.mdpi.com/article/10.3390/nu18142301/s1, File S1: Selection of sites based on composite indicators; Table S1: Sample size and sampling method; Table S2: Analysis of determinants of compliance to IFA in the endline survey based on the output indicators as described in the Program Impact Pathway (PIP); Table S3: Improvement in programmatic activities during the intervention period.

## Abbreviations

Abbreviation	Full Form
IFA	Iron and Folic Acid
AMB	Anemia Mukt Bharat
NI	Nutrition International
PW	Pregnant Woman
LW	Lactating Woman
FLW	Frontline Worker(s)
ASHA	Accredited Social Health Activist
ANM	Auxiliary Nurse Midwife
AWW	Anganwadi Worker
MO	Medical Officer
ANC	Antenatal Care
PNC	Postnatal Care
VHSND	Village Health, Sanitation and Nutrition Day
PHC	Primary Health Center
Hb	Hemoglobin
IEC	Information, Education and Communication
PIP	Project Impact Pathway
DID	Difference-in-Difference
OR	Odds Ratio
aOR	Adjusted Odds Ratio
CI	Confidence Interval
MP	Madhya Pradesh
CSPro	Census and Survey Processing System
IRB	Institutional Review Board
