# Supplementary material for: Effectiveness of Health Systems Strengthening Implementation Model to Improve Health of Pregnant and Lactating Women by Increasing Compliance with IFA and Calcium Tablets: A Quasi-Experimental Study
Source: Nutrients. 2026 Jul 14;18(14):2301. doi: 10.3390/nu18142301 (PMC13416310; doi:10.3390/nu18142301)
Supplement: Supplementary file 1 [file nutrients-18-02301-s001.zip › nutrients-4356334-supplementary files.pdf]

Title: Effectiveness of health systems strengthening implementation model to improve compliance to IFA and Calcium tablets in pregnant and lactating women- a quasi experimental study

Supplementary File S1: Selection of sites based on composite indicators

The study was undertaken in two states of India (Gujarat and Madhya Pradesh). The following steps were followed for selection of study blocks:

1) **Review of indicators:** Indicator is a measurable element of practice performance for which there is evidence or consensus that it can be used to assess the quality or performance. The indicators were reviewed based on extent to which they accurately represent the concept being assessed.
2) **Verification of availability of data district wise in both the states (Gujarat & MP)**
After selection of the indicators most suitable to decide on the study site, the availability of data against each indicator has been verified from various sources. Data for some indicators was not available district wise, and those indicators have been removed from the process.
3) **Finalization of indicators**
After removing some indicators, list of following 16 indicators have been finalized and categorized in the three sections.
    a) Indicators related to maternal nutritional status
    b) Indicators related to Health system / infrastructure
    c) Indicators related to socio-demographic development

Indicators related to maternal nutritional status

a) Early registration of pregnancy
b) Pregnant women consuming IFA for 100 days or more
c) Women receiving 4 or more antenatal care check-ups to the total no. of pregnant women registered for antenatal care
d) Pregnant women receiving IFA supplementation (180 IFA tablets)
e) Pregnant women aged 15-49 years who are anaemic (<11.0 g/dl) (%)
f) Pregnant women receiving Calcium supplementation (360 tablets)
g) Severely anemia pregnant women (Hb<7) treated at institution
h) Complicated Pregnancies treated with Blood Transfusion in facilities
i) Proportion of institutional deliveries
j) Proportion of home deliveries attended by an SBA (Skilled Birth Attendance)

Indicators related to Health system / infrastructure

a) Proportion of Sub centres converted into Health & Wellness Centres (HWCs)
b) Proportion of PHCs converted into Health & Wellness Centres (HWCs)

Indicators related to socio-demographic development

a) Female Literacy rate %
b) Households with an improved drinking-water source
c) Households using improved sanitation facility (%)

d) Household with electricity (%)

4) **Collection of data against all the indicators from various reliable resources**
On the above mentioned indicators, data have been collected from various reliable secondary sources. The data sets such as HIMS, NFHS, Census and official websites of the districts have been used.

5) **Making of composite index based on separate indices of all the indicators selected.**
For each indicator, separate index has been formulated with the help of following formula

For the indicators for which higher values are better (Positive Indicator), following formula has been used.

Index of Positive Indicator =$(Xi - minimum\ value)/\ (maximum\ value - minimum\ value)$

Similarly, for indicators for which lower values are better, following formula has been used.

Index of Negative Indicator =$(\ Maximum\ value - Xi)/\ (maximum\ value - minimum\ value)$

After Individual indices, three different indices have been made by taking simple arithmetic average based on three categories. Thereafter, for each category we got one index and by taking simple arithmetic mean further, we got one composite index district wise for both the states (Gujarat & MP) values were distributed between 0 and 1, with the best performing district at 1 and the worst performing district at 0.

6) **Selection of the district**
Four districts from each state have been selected based on the composite index value around the median indices. Selection of the districts also took into consideration the similar programs already running in the district to avoid overlapping in the outcome to select one district.

7) **Selection of blocks**
After the selection of the districts two blocks were selected from each district. Based on the indicators mentioned above, one high performance block and one low performance block were selected.

Supplementary Table S1: Sample size and sampling method

Sample size:

**Assumptions**	**Round 1 survey (2022)**	**Round 2 survey (2024)**
Respondent group : Pregnant and lactating women of reproductive age		
Assumed Design effect	1.5	1.5
Assumed value of the indicator P1 (expressed as proportion/ fraction of one) (Mothers who consumed iron folic acid for 100 days or more when they were pregnant- Source: NFHS-4). This value varies from 23.6% in case of Madhya Pradesh to 36.8% in case of Gujarat. An average of the state values is considered here as 30.2%.	0.302	0.302
Assumed value of the indicator P2 assuming a 10%-point change/ difference (expressed as proportion/ fraction of one)	0.402	0.402
$Z\alpha$ (95% confidence level)	1.645	1.645
$Z\beta$ (80% power)	0.842	0.842
**Program areas**		
Currently pregnant women 15 to 49 years of age in the community (100 clusters/ villages x 5 pregnant women per cluster) in four blocks (25 clusters per block) considering the difficulty in getting the required no. of PW in a cluster	500	500
Lactating women (0 to 5 months post-partum) 15 to 49 years of age in the community (100 clusters/ villages x 5 lactating women per cluster) in four blocks (25 clusters per block) considering the difficulty in getting the required no. of LW in a cluster	500	500
Frontline health functionaries (ANMs + AWWs + ASHAs)	150	150
**Comparison areas**		
Currently pregnant women 15 to 49 years of age in the community (100 clusters/ villages x 5 pregnant women per cluster) in four blocks (25 clusters per block) considering the difficulty in getting the required no. of PW in a cluster	500	500
Lactating women (0 to 5 months post-partum) 15 to 49 years of age in the community (100 clusters/ villages x 5 lactating women per cluster) in four blocks (25 clusters per block) considering the difficulty in getting the required no. of LW in a cluster	500	500
Frontline health functionaries (ANMs + AWWs + ASHAs)	150	150
Total	**2,300**	**2,300**

Sampling:

Selection of PHC: The list of primary health centres (PHCs) by different blocks were prepared and arranged in ascending order based on population size of village. After arranging the list of PHCs by their population coverage at village level, random numbers were generated between lowest and highest size of the population. Those PHCs were selected for all four Blocks/Taluks where the size of the village population was equal or just

greater than random number generated.

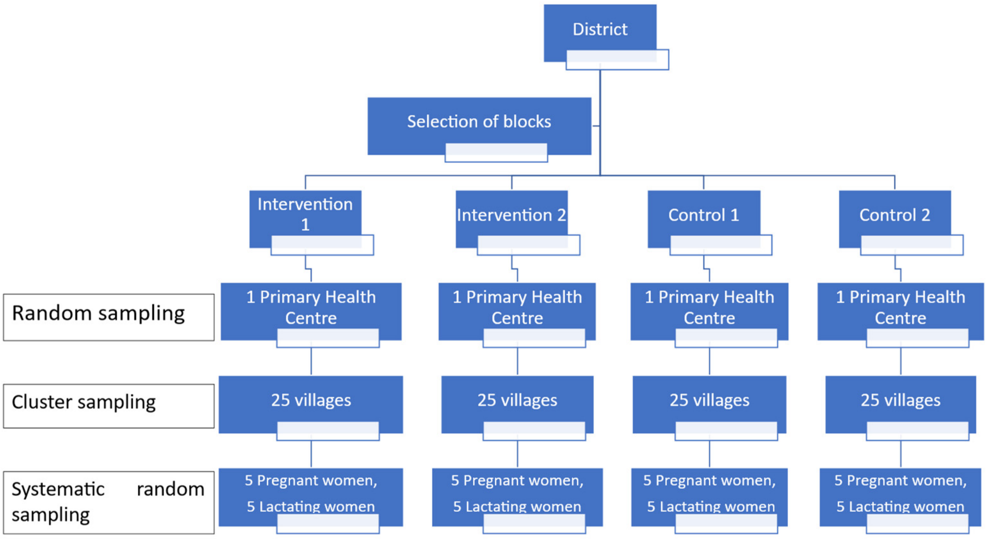

Figure S1: A flowchart of the study design and sample collection

Selection of villages: The list of villages by each sub-centres against each primary health centres were arranged in ascending order. The lower and upper interval of population proportion were computed after computation of cumulative population proportion at village level. The village was selected using random number method in Microsoft excel. The process was repeated until desired number of villages get selected. Some of the villages were selected multiple times due to higher probability of selection of village (size) at PHC level.

Table S1.1: List of sampled Primary Health Centre in selected states of India

**Selected PHCs in Madhya Pradesh, India**		
**Taluk/ Block**	**Selected PHC**	**Total number of selected PHCs**
Damoh	Bandhakpur	**4**
Patharia	Jerath	
Patera	Kumhari	
Tedukheda	Sarra	
**Selected PHCs in Gujarat, India**		
Dabhoi	Thuvavi	**4**
Desar	Navasihora	
Savli	Bhadrva	
Vadodara	Bhaili	

Selection of pregnant women: From the centre of the village, a direction was selected randomly and followed. Every 5thhousehold was visited to identify pregnant and lactating women fitting the inclusion criteria. The process continued till 5 pregnant and 5 lactating women were surveyed.

The inclusion criteria included women who:

- were residents of the same village
- If pregnant, they were in second or third trimester
- were willing to give consent and participate in interviews

Houses which were locked at the time of the visit, as well as houses where the mother was not present/ unable to participate on the day of the visit was excluded. In cases both pregnant and lactating women were present in the same household, only one was considered for the survey. In case there were multiple floors of the same building, all the floors were considered provided they did not belong to the same family. For each excluded household, the reason for exclusion was noted.

Supplementary Table S2: Analysis of determinants of compliance to IFA in the endline survey based on the output indicators as described in the Program Impact Pathway (PIP)

**Indicators**	**Baseline coverag**	**Endline coverage**	**Association of endline coverage with compliance**		
			OR	95% CI	P value
**Compliance to IFA in endline survey (both states combined)**	31.5%	36.5%			
1. **Improved counselling skills of FLWs**					
Respondents who were informed about anemia status (out of those Hb was tested)	**24.84 (236)**	41.41 (381)	1.15	1.14; 2.0	0.003
Respondents who were informed about Hb level (out of those Hb was tested)	57.79 (549)	68.98 (636)	0.76	0.58; 1.0	0.06
Respondents who were informed about IFA intake (out of those Hb was tested)	93.9 (969)	97.69 (931)	0.5	0.19; 1.25	0.14
Respondents correctly advised to consume 2 tabs of IFA (out of those who were informed about their Anemic status)	24.5 (182)	38.88 (243)	2.45	1.47; 4.06	0.001
Counselling by ANMs	43.9 (453)	34.7 (331)	0.57	0.43; 0.76	0.001
Counselling by ASHAs	18.8 (194)	38.4 (366)	1.2	0.98; 1.68	0.06
Counselling by AWWs	11.43 (118)	8.5 (81)	1.39	0.50; 3.44	0.001
Counselling by MOs	24.1 (249)	17 (162)	1.3	0.9; 1.8	0.16
Counselling about benefits of consuming IFA	62.06 (651)	82.9 (795)	1.46	1.01; 2.11	0.04
Counselling about dosage	72.45 (760)	66.1 (633)	1.66	1.25; 2.22	0.001
Counselling about side effects	3.15 (33)	13.6 (33)	1.17	0.84; 1.63	0.3
Improved knowledge and awareness of beneficiaries					
2. **Instructions given while consuming IFA**					
Consume the IFA tablet about 1 hour after proper food	**43.28(454)**	83.51 (800)	2.59	1.72; 3.93	0.001
Not to consume in empty stomach	45.66(479)	56.05 (537)	1.01	0.78; 1.32	0.913
To be consumed in the Morning	51.48(540)	45.41 (435)	0.55	0.42; 0.73	0.001
To be consumed in the Evening	38.32(402)	40.61 (389)	0.75	0.57; 0.98	0.039
Drink plenty of water during the day	9.06(95)	13.67 (131)	1.12	0.77; 1.64	0.54
Consume lots of fruits and vegetables to avoid constipation	7.72(81)	8.35 (80)	1.47	0.93; 2.33	0.102
Do not consume tablet(s) with tea, coffee or milk	1.53(16)	4.8 (46)	1.63	0.90; 2.95	0.106
If severe symptoms appear, consult doctor immediately	0.29(3)	1.88 (18)	1.4	0.55; 3.58	0.484
IFA supplement with Nimbu-pani	3.24(34)	14.82 (142)	1.86	1.29; 2.66	0.001
Consume citrus fruits or Achar or Imli with IFA supplements	1.53(16)	142 (70)	1.41	0.87; 2.32	0.164
Do not consume with Calcium (CA)	5.82(61)	8.04 (77)	1.77	1.11; 2.84	0.016
No instructions given	4.86(51)	2.71 (26)	0.31	0.10; 0.90	0.032

**Women who had heard of the word 'anemia" (out of all pregnant women)**	**6.69 (69)**	**60.02 (572)**	**1.25**	**0.96; 1.64**	**0.097**
Women who knew what is anemia	45.59 (31)	70.73 (406)	0.83	0.57; 1.21	0.34
Women who knew about signs and symptoms of anemia	5.62 (59)	57.72 (553)	0.75	0.58; 0.99	0.041
Women knew how to prevent anemia	98.47(1033)	97.49 (934)	1.75	0.69; 4.44	0.24
3. **Improved supply chain**					
Received more than 30 tablets received in the last visit	**82.65(867)**	87.68 (840)	1.72	1.11; 2.64	0.014
Received more than 90 tablets received in the last 3 visits	85.42(895)	93.11 (892)	0.93	0.71; 1.21	0.598
4. **Engagement of family members**					
Care by husbands (accompanying for ANC, iron rich food, household chores)	92.18(967)	98.75 (946)	2.91	0.63; 13.3	0.17
Care by family members (accompanying for ANC, iron rich food, household chores)	88.85(932)	95.82 (918)	2.81	1.23; 6.43	0.014
5. **Quality of ANC services**					
Registration of pregnancies	**98.35 (1015)**	99.58 (950)	1.76	0.25; 12.5	0.56
Visited Government facilities for ANC	76.94 (794)	82.37 (785)	0.8	0.57; 1.13	0.205
Quality of ANC services (as informed by respondents)					
Weight measured	96.6 (966)	99.27 (946)	3.42	0.41; 28.6	0.255
Weight measured and recorded	86.72 (895)	93.6 (892)	1.27	0.73; 2.23	0.397
Hb measured	92.5 (925)	96.33 (918)	1.33	0.67; 2.65	0.405
Hb measured and recorded	90.19 (855)	91.28 (848)	0.72	0.43; 1.18	0.188
BP measured	95.6 (956)	98.95 (943)	2.29	0.48; 10.8	0.297
BP measured and Recorded	84.59 (873)	93.91 (895)	1.39	0.78; 2.49	0.262
USG done	61.1 (611)	72.09(687)	1.73	1.27; 2.36	0.001
Blood sugar	70.4 (704)	91.18 (868)	2.09	1.23; 3.54	0.006
All services provided (Wt, Hb, BP, USG, BS)	51.16(528)	**69.46 (662)**	**1.89**	**1.39; 2.56**	**0.001**
Received IFA from VHSND	56.05(588)	68.27 (654)	1.11	0.83; 1.47	0.465
Received IFA from AWC	1.33(14)	22.03 (211)	1.26	0.92; 1.72	0.149

Supplementary Table S3: Improvement in programmatic activities during the intervention period

**Proportion of pregnant/ lactating women who came in contact with ASHAs during pregnancy**					
		Pregnant women		*Lactating women*	
		Baseline	***Endline***	***Baseline***	***Endline***
**Both states**		53.19 (n = 1049)	85.59* (n = 958)	32.57 (n = 1053)	59.59* (n = 1064)
**MP (Damoh)**		56.44 (n = 528)	89.31* (n = 505)	20.98 (n = 529)	68.12* (n = 549)
**Gujarat (Vadodara)**		49.9 (n = 521)	89.31* (n = 453)	44.27 (n = 524)	54.9*(n = 515)
**Both states**	Control	56.48 (n = 517)	84.86* (n = 436)	33.53 (n = 519)	67.74* (n = 468)
	Intervention	50 (n = 532)	86.21* (n = 522)	31.65 (n = 534)	53.9* (n = 596)
**MP (Damoh)**	Control	58.24 (n = 261)	86.56* (n = 253)	20.53 (n = 263)	69.18* (n = 279)
	Intervention	54.68 (n = 267)	92.06* (n = 252)	69.18 (n = 266)	67.04* (n = 270)
**Gujarat (Vadodara)**	Control	54.69 (n = 183)	82.51* (n = 256)	46.88 (n = 256)	65.61* (n = 189)
	Intervention	45.28 (n = 270)	80.74* (n = 265)	41.79 (n = 268)	41.72 (n = 326)
**Distribution of IFA tablets in adequate quantities**					
**Both states**		38.4 (n = 1049)	61.59* (n = 958)	35.14 (n = 1053)	50.75*(n = 1064)
**MP (Damoh)**		45.45 (n = 528)	78.81* (n = 505)	41.02 (n = 529)	73.41* (n = 549)
**Gujarat (Vadodara)**		31.29 (n = 521)	42.38* (n = 453)	29.2 (n = 524)	26.6 (n = 515)
**Both states**	Control	38.49 (n = 517)	58.72* (n = 436)	26.4 (n = 519)	55.77* (n = 468)
	Intervention	38.35 (n = 532)	63.98* (n = 522)	43.63 (n = 534)	46.81 (n = 596)
**MP (Damoh)**	Control	47.51 (n = 261)	73.91* (n = 253)	31.94 (n = 263)	69.53* (n = 279)
	Intervention	43.45 (n = 267)	83.73* (n = 252)	50.0 (n = 266)	77.41* (n = 270)
**Gujarat (Vadodara)**	Control	29.3 (n = 183)	37.7 (n = 256)	20.7 (n = 256)	35.45* (n = 189)
	Intervention	33.21 (n = 270)	45.56* (n = 265)	37.31 (n = 268)	21.47* (n = 326)
**Distribution of Calcium tablets in adequate quantities**					
**Both states**		14.78 (n = 1049)	44.57* (n = 958)	10.73 (n = 1053)	44.08* (n = 1064)
**MP (Damoh)**		19.89 (n = 528)	60.2* (n = 505)	10.73 (n = 529)	44.08* (n = 549)
**Gujarat (Vadodara)**		9.6 (n = 521)	27.15* (n = 453)	7.06 (n = 524)	23.5* (n = 515)
**Both states**	Control	15.67 (n = 517)	42.2* (n = 436)	5.39 (n = 519)	48.72* (n = 468)
	Intervention	13.91 (n = 532)	46.55* (n = 522)	15.92 (n = 534)	40.44* (n = 596)
**MP (Damoh)**	Control	25.67 (n = 261)	54.94* (n = 253)	8.37 (n = 263)	58.78* (n = 279)
	Intervention	14.23 (n = 267)	65.48* (n = 252)	20.3 (n = 266)	68.15* (n = 270)
**Gujarat (Vadodara)**	Control	29.3 (n = 183)	37.7 (n = 256)	20.7 (n = 256)	35.45* (n = 189)
	Intervention	33.21 (n = 270)	45.56* (n = 265)	37.31 (n = 268)	21.47* (n = 326)
**Proportion of women who underwent Hb estimation during pregnancy**					
**Both states**		90.37 (n = 1049)	95.82* (n = 958)	89.55 (n = 1053)	88.82(n = 1064)
**MP (Damoh)**		89.39 (n = 528)	95.05* (n = 505)	90.74 (n = 529)	91.07 (n = 549)
**Gujarat (Vadodara)**		91.36 (n = 521)	96.69* (n = 453)	88.36 (n = 524)	86.41(n = 515)
**Both states**	Control	94.36(n = 517)	96.33 (n = 436)	95.76 (n = 519)	87.18* (n = 468)
	Intervention	86.09 (n = 532)	95.4* (n = 522)	83.52 (n = 534)	90.1* (n = 596)
**MP (Damoh)**	Control	93.49 (n = 261)	96.05 (n = 253)	96.96 (n = 263)	91.76* (n = 279)
	Intervention	85.39 (n = 267)	94.05* (n = 252)	84.59 (n = 266)	90.37* (n = 270)
**Gujarat (Vadodara)**	Control	96.09 (n = 183)	96.72 (n = 256)	94.53 (n = 256)	80.42* (n = 189)
	Intervention	86.79 (n = 270)	96.67* (n = 265)	82.46 (n = 268)	89.88* (n = 326)
**Prevalence of severe anemia**					
**Both states**		**3.53 (n = 1049)**	**2.92(n = 958)**	-	-

**MP (Damoh)**		4.92 (n = 528)	4.95 (n = 505)	-	-
**Gujarat (Vadodara)**		2.11 (n = 521)	0.66(n = 453)	-	-
**Both states**	Control	2.90(n = 517)	4.14 (n = 436)	-	-
	Intervention	3.67 (n = 532)	2.30 (n = 522)	-	-
**MP (Damoh)**	Control	3.45 (n = 261)	6.37 (n = 253)	-	-
	Intervention	5.14 (n = 267)	4.76 (n = 252)		
**Gujarat (Vadodara)**	Control	2.34 (n= 183)	1.64 (n=256)		
	Intervention	1.89 (n=270)	0.05* (n=265)	-	-

*difference between baseline and endline indicators $p<0.05$
